# Activation and clinical significance of the unfolded protein response in breast cancer

**DOI:** 10.1038/sj.bjc.6605365

**Published:** 2009-10-27

**Authors:** P Scriven, S Coulson, R Haines, S Balasubramanian, S Cross, L Wyld

**Affiliations:** 1 Academic Unit of Surgical Oncology, Department of Oncology, School of Medicine, Dentistry and Health, University of Sheffield, Beech Hill Road, Sheffield S10 2JP, UK; 2 Academic Unit of Pathology, Department of Neuroscience, School of Medicine, Dentistry and Health, University of Sheffield, Beech Hill Road, Sheffield S10 2RX, UK

**Keywords:** breast cancer, unfolded protein response, stress response

## Abstract

**Introduction:**

The tumour microenvironment is hypoglycaemic, hypoxic and acidotic. This activates a stress signalling pathway: the unfolded protein response (UPR). The UPR is cytoprotective if the stressor is mild, but may initiate apoptosis if severe.

Activation of the UPR in breast carcinoma is induced by microenvironmental stress such as glucose and oxygen deprivation, but may also be linked to oestrogen stimulation. It may be clinically significant as it may alter chemosensitivity to doxorubicin.

**Methods:**

395 human breast adenocarcinomas were immunohistochemically stained for UPR activation markers (glucose-regulated protein (GRP-78 and XBP-1). A model of UPR activation *in vitro* by glucose deprivation of T47D breast cancer cells was developed to determine how the UPR affects cellular sensitivity to doxorubicin and 5-fluorouracil. Cytotoxicity was assessed using a colorimetric cytotoxicity assay (MTT). The effect of oestrogen stimulation and tamoxifen exposure on UPR activation by T47D cells was determined by western blotting measurement of the key UPR protein, GRP-78.

**Results:**

Expression of GRP78 and XBP-1 was demonstrated in 76% and 90% of the breast cancers, respectively, and correlated with oestrogen receptor positivity (*P*=0.045 and 0.017, respectively). *In vitro* UPR activation induced resistance to both doxorubicin and 5-flurouracil, (*P*<0.05). Oestrogen stimulation induced GRP78 and XBP1 over-expression on western blotting. Tamoxifen did not block this response and may induce UPR activation in its own right.

**Conclusions:**

The UPR is activated in the majority of breast cancers and confers resistance to chemotherapy. *In vitro* oestrogen stimulates UPR induction. UPR activation may contribute to breast cancer chemoresistance and interact with oestrogen response elements.

The unfolded protein response (UPR), is a stress response pathway whose physiological function is to safeguard the synthesis of normal cellular proteins (Scriven *et al*, 2007a). Protein synthesis in the endoplasmic reticulum is a vital component of cellular activity. Controlling the quality of protein products exported from the endoplasmic reticulum to the remainder of the cell is critical to ensuring normal cellular function (Hammond and Helenius, 1994). Proteins are synthesised initially as simple, linear, polypeptide chains and subsequently undergo a complex glycosylation and folding process before assuming the correct functional conformation. Folding is an energy-dependent process and incorrect folding or glycosylation may occur in the presence of glucose deprivation. This leads to the accumulation of malfolded or incompletely folded proteins in the endoplasmic reticulum. These ‘unfinished’ proteins are bound to chaperone molecules such as glucose-regulated protein 78 (GRP 78) to prevent their export from the endoplasmic reticulum whence they might disrupt cellular function (Wei and Hendershot, 1995). The cell has mechanisms to detect increased levels of malfolded proteins and will then initiate the UPR (Kozutsumi *et al*, 1988).

This UPR pathway has wide ranging cellular effects including (Scriven *et al*, 2007a):

1. Reduced global protein synthesis and cell-cycle arrest.
2. Activation of UPR responsive genes.
3. Inhibition of apoptosis if the stress is mild or reversible.
4. Initiation of apoptotic pathways if the stress is severe or prolonged.

Human solid tumours contain areas of poor vascular perfusion, which result from inadequacies in the tumour microcirculation and cancer cells preferentially use glucose as an energy source – the Warburg effect (Warburg, 1956). The net result is that human tumours typically exist in an environment of glucose and oxygen deprivation. Although tumour cells may undergo necrosis or apoptosis if these conditions are severe, they have cellular survival strategies, which enable them to withstand these severe conditions (for example induction of angiogenesis and the UPR). Under conditions of mild to moderate stress, the UPR is anti-apoptotic and enables cells to withstand such stressful stimuli and it has recently been shown that UPR induction is vital for allowing cells to withstand periods of hypoxia (Koumenis and Wouters, 2006). This ability of the UPR to protect cells (including cancer cells) from adverse conditions underpins its clinical significance.

The UPR is potentially important in the field of oncology for a number of reasons. The UPR is activated in a range of human solid tumours including breast cancer (Fernandez *et al*, 2000), gastric cancer (Zhang *et al*, 2006; Scriven *et al*, 2007b), hepatocellular carcinoma (Shuda *et al*, 2003; Al-Rawashdeh *et al*, 2008), pancreatic cancer (Scriven *et al*, 2008) and lung cancer (Uramoto *et al*, 2005).

A link has been shown between UPR activation and poor clinical outcome, with glucose-deprived tumours having increased metastatic potential (Schlappack *et al*, 1991). High levels of GRP78 expression correlate with increasing tumour grade in hepatocellular carcinoma (Shuda *et al*, 2003), poor clinical outcome in breast cancer (Lee *et al*, 2006), higher recurrence and mortality rates in prostate cancer (Daneshmand *et al*, 2007) and a higher rate of nodal metastasis and reduced survival in gastric cancer (Zhang *et al*, 2006).

Over-expression of the UPR may also be clinically important because it reduces the efficacy of certain types of chemotherapy, such as doxorubicin (Shen *et al*, 1987; Hughes *et al*, 1989; Chatterjee *et al*, 1995), one of the mainstays of most breast cancer chemotherapy regimens. The UPR also interacts with oestrogen responsive pathways in breast cancer and has recently been proposed to contribute to the development of anti-oestrogen resistance (Fu *et al*, 2007; Gomez *et al*, 2007; Davies *et al*, 2008).

It is therefore likely that the high level of UPR activation in human breast cancers will be of clinical significance, because of the inhibition of chemosensitivity and its potential role in the development of anti-oestrogen resistance. The aims of this study were to further clarify these associations.

## Materials and methods

### Materials

Anti-GRP78 goat polyclonal antibody (sc-1050, Santa Cruz Biotechnology Inc., Santa Cruz, CA, USA), anti-XBP-1 rabbit polyclonal antibody (cat no. 619501, BioLegend, San Diego, CA, USA), Vectastain ABC elite goat and rabbit kits (Vector Laboratories Inc., Burlingame, CA, USA), anti-GRP78 mouse monoclonal antibody (cat no 610979, BD Biosciences, San Jose, CA, USA), anti-XBP-1 mouse polyclonal antibody (H7494-B01, Abnova, Taipei, Taiwan) and recombinant positive control H7494-P01 (54 kDa), anti-hypoxia inducible factor-1*α* (HIF-1*α*) mouse monoclonal antibody (cat no 61095, BD Biosciences) *β*-actin mouse monoclonal antibody (Sigma Aldrich Company Ltd, Dorset, UK), RPMI 1640 and glucose-free RPMI 1640 media (Invitrogen Corporation, Carlsbad, CA, USA), dialysed fetal bovine serum (FBS) (Sigma Aldrich Company Ltd), penicillin and streptomycin (Lonza Group Ltd, Basel, Switzerland), D-glucose anhydrous (Sigma Aldrich Company Ltd), 5-fluorouracil (Sigma Aldrich Company Ltd), doxorubicin hydrochloride (Sigma Aldrich Company Ltd), 17*β*-oestradiol (Sigma Aldrich Company Ltd).

### Immunohistochemistry

#### Sample collection and preparation

Appropriate ethical committee approval was obtained (SSREC/02/155 and SSREC 04/Q2305/67); 395 cases of breast cancer were identified. The tumour areas were identified and sampled in triplicate with a 0.6 mm diameter tissue array punch (Beecher Instruments Inc., Sun Prairie, WI, USA) to create a tissue micro array. Each case was given a unique identifier and linked to a database containing clinico-pathological data. The completed array contains 296 ductal, 39 lobular, 21 tubular and 39 other histological types.

Ten cases of DCIS were identified and sequential sections cut from a formalin-fixed paraffin-embedded sample and stained for GRP78, XBP1 and the marker of hypoxia HIF-1*α*.

#### Immunohistochemistry method

Staining for GRP78 and XBP-1 proteins was performed using the avidin–biotin–peroxidase complex method (Hsu *et al*, 1981). For antigen retrieval the sections where immersed in 0.01 M tris–sodium citrate adjusted to pH 6.0 and boiled for 10 min followed by 10 min simmering at 75°C. Slides were incubated with anti-GRP78 or anti-XBP1 primary antibody (both at 1 : 100) for 30 min at room temperature and then washed in phosphate-buffered saline. Biotinylated anti-goat or anti-rabbit IgG was used as a secondary antibody and visualised with diaminobenzidine. For each antibody, three slides were stained representing triplicate samples from each case and scored semi-quantitatively: 0 negative, 1 weak positive, 2 intermediate positive and 3 strongly positive. Scores were totalled across the three cores; cases with only one core present were excluded, cases with two viable scores had the third missing score replaced with the mean of the two viable cores. Scoring was performed by SSC – an experienced breast disease histopathologist and PS. The correlation coefficient for scoring of cores between the two observers was high (GRP78 *r*=0.978, for XBP1 *r*=0.956).

For HIF-1*α* staining on the DCIS sections antigen retrieval used the DAKO Target retrieval solution and the DAKO Catalysed Signal Amplification kit. Three sequential sections of each case were stained for GRP78, XBP1 and HIF-1*α*.

### *In vitro* studies

#### Validation and method of *in vitro* model of UPR induction

##### Cell culture

The human breast cancer cell line T47D was obtained from the European Collection of Animal Cell Cultures (ECACC) and maintained in RPMI 1640 with 10% dialysed FBS, 1% L-glutamine and 1% penicillin and streptomycin at 37°C in 5% CO_2_ humidified air. For glucose deprivation experiments glucose-free RPMI 1640 was supplemented with D-glucose to yield media with glucose concentrations of 1, 0.5 and 0.2 mmol. Cells were seeded into standard T75 culture flasks and allowed to settle for 24 h in standard media. The media was then exchanged for glucose-deprived media and flasks left for 24, 48 and 72 h before lysing cells for western blotting.

For oestrogen stimulation experiments, cells were grown in standard media supplemented with 0.5, 1 and 5 nM of 17*β*-oestradiol. One nanomolar 17*β*-oestradiol represents a concentration that would be expected in a normal premenopausal female and has previously been shown to induce GRP78 (Gilligan *et al*, 1994; Kiang *et al*, 1997) Flasks were lysed after 24 and 48 h exposure.

Tamoxifen was dissolved in DMSO to yield a 1 mM solution, which was then serially diluted with RPMI 1640 media to yield working concentrations of 1, 10 and 50 *μ*M tamoxifen in 0.1% DMSO; 0.1% DMSO does not have any effect on the growth of T47D cells (Lim *et al*, 2006). Cells were exposed for 24 and 48 h before lysing.

##### Western blot analysis

Cells were lysed in triple detergent lysis buffer (0.1% sodium dodecyl sulphate, 1% nonidet P-40, 0.5% sodium deoxycholate) and complete EDTA-free protease inhibitor cocktail (F. Hoffmann-La Roche AG, Basel, Switzerland) on ice and centrifuged at 13 000 r.p.m. for 10 min to remove insoluble material. The supernatant was stored at −80^o^C. Whole cell lysates and recombinant GRP78 protein as a positive control (gifted by Dr Valerie Corrigal, Kings College London) were electrophoretically separated through a 10% SDS–polyacrylamide gel and blotted onto PVDF membrane (Millipore Inc., Billerica, MA, USA). Antibodies for western blotting were anti-GRP78 (BD Biosciences) 1 : 1000, anti-XBP1 detecting the 54 kDa spliced variant (Abnova) 1 : 1000 and anti-*β*-actin 1 : 10 000. Appropriate horseradish peroxidase-conjugated IgGs were used as secondary antibodies. Blots were developed with SuperSignal West Dura Extended Duration Substrate (Thermo Scientific Inc., Waltham, MA, USA) and imaged with a Syngene CCD imaging system (Synoptic Ltd, Cambridge, UK). Bio-Rad Quantity One (Bio-Rad Laboratories Inc., Hercules, CA, USA) software was used on the raw image to allow semi-quantitation of blot signal intensity relative to the actin signal intensity. A minimum of three blots for each experimental arm was performed.

##### Chemosensitivity assay

The colourimetric thiazolyl blue tetrazolium bromide (MTT) assay was used to determine response to chemotherapy agents. Reduction of MTT by mitochondrial dehydrogenase enzymes produces purple formazan crystals, which can be solubilised by DMSO and quantified by absorbance photometry. An optimum seeding density of 37 500 per cells cm^−2^ was determined and 96-well plates were seeded accordingly. Inhibitory concentration 50 (IC_50_) doses of doxorubicin and 5-FU were determined for cells in standard culture conditions. Subsequently 96-well plates were seeded and after 24 h the media exchanged for glucose-deprived media and a further 24 h later the IC_50_ dose of either doxorubicin or 5-FU applied and the plate read 48 h later. The relative percentage viability for each glucose concentration was then calculated. Each arm comprised three triplicate repeats.

##### Statistical analysis

Statistical analysis was performed using SPSS version 15 (SPSS Inc., Chicago, IL, USA) Statistical significance was set at *P*<0.05.

## Results

### Immunohistochemistry

**Figure 1:**
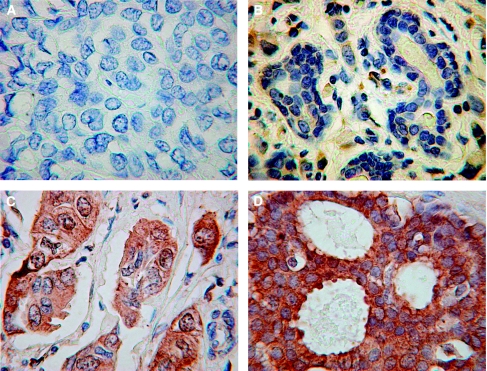
Representative photmicrographs of GRP78-stained breast tissue cores from the TMA (magnification × 40). (**A**) Negatively stained normal breast tissue, (**B**) weakly stained core score 1, (**C**) intermediately stained core scores 2, (**D**) strongly stained core scores 3.

Of the *ex-vivo* breast cancer samples on the tissue micro array, 76% stained strongly (score >6/9) for GRP78 and 90% stained strongly for XBP1. Normal tissue on the array did not stain for either protein. Figure 1 shows representative sample cores of non-staining, weakly staining, intermediate and strongly staining cores. GRP78 is principally distributed within the cytoplasmic regions, XBP1 is distributed in both the cytoplasmic and nuclear regions of cells, as would be expected from their known biological mechanisms of action (Scriven *et al*, 2007a).

**Figure 2:**
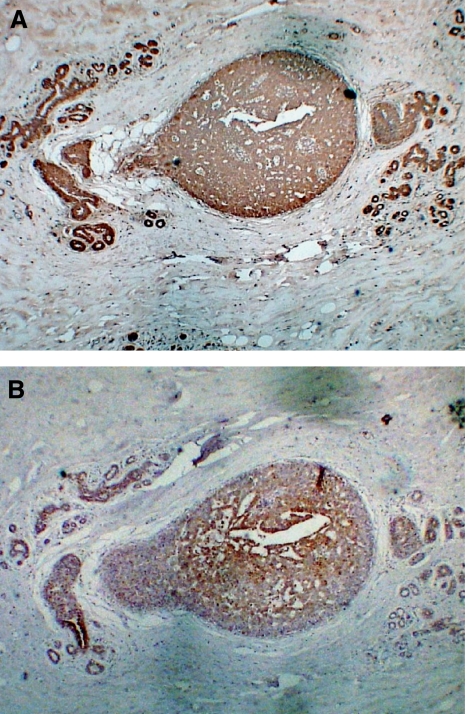
Representative photomicrographs of DCIS stained for GRP78, HIF-1*α* and XBP1 (magnification × 40). Immunohistochemical staining of a representative DCIS specimen. (**A**) Is stained for GRP78 a marker of UPR activation and (**B**) is stained for HIF-1*α*, a marker of response to hypoxia.

All 10 DCIS sections stained positive in tumour regions for GRP78, 8 of 10 had detectable nuclear XBP1 staining. Adjacent normal breast tissue did not stain for either GRP78 or XBP1 in keeping with the findings of the tissue micro array. HIF-1*α* staining was observed in seven samples of which all showed co-expression in tumour regions with GRP78 and six showed co-expression of all three proteins in tumour regions. (Figure 2 shows a representative section stained for GRP78 and HIF-1*α*).

High GRP78 and XBP1 scores were associated with oestrogen receptor (ER) positivity (*P*=0.045 and 0.017, respectively). There was no statistically significant correlation with tumour size, grade, lymph node metastases, vascular invasion, histological type or Nottingham Prognostic Index (Haybittle *et al*, 1982).

High GRP78 scores correlated with high XBP1 scores *P*=<0.01 (Spearman's correlation coefficient).

### *In vitro* model of UPR induction by glucose deprivation

**Figure 3:**
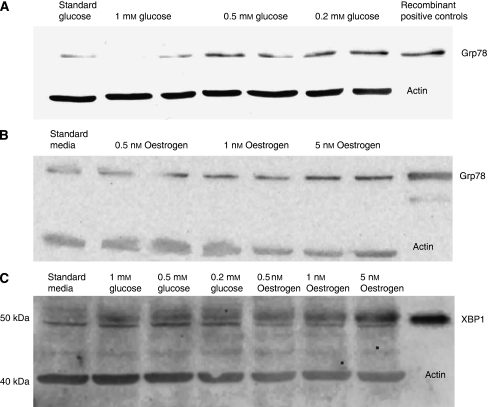
Induction of GRP78 and XBP1 by glucose deprivation and oestrogen stimulation. Western blots of breast cancer cell line lysates (T47D) after exposure to varying culture conditions to demonstrate GRP78 or XBP1 expression. (**A**) Cells cultured in differing glucose concentrations varying from normal to 0.2 mM showing progressively increasing GRP78 levels as glucose concentrations fall. (**B**) Cells cultured in progressively increasing oestrogen concentrations showing GRP78 over-expression. (**C**) Cells cultured in varying glucose or oestrogen concentrations showed that lack of glucose or oestrogen stimulation induced XBP1 expression.

Culture under conditions of progressive glucose deprivation for 24 and 48 h induced expression of GRP78 on western blotting (Figure 3A). XBP1 is detectable in all glucose-deprived samples but not in lysates from standard culture conditions (Figure 3C). Analysis of raw image data using Bio-Rad Quantity One for a minimum of three blots confirms increasing signal density in glucose-deprived lysates compared with normal controls.

### *In vitro* oestrogen stimulation

Exposure to 0.5, 1 and 5 nM 17-*β*-oestradiol for 24 and 48 h induces GRP78 expression in a dose-dependant manner compared with standard non-stimulated cells (Figure 3B). XBP1 is detectable in all oestrogen-stimulated samples but not in lysates from standard culture conditions (Figure 3C). Analysis of raw image data using Bio-Rad Quantity One for a minimum of three blots confirms increasing signal density in oestrogen-stimulated lysates compared with normal controls.

### The *in vitro* effect of tamoxifen

**Figure 4:**
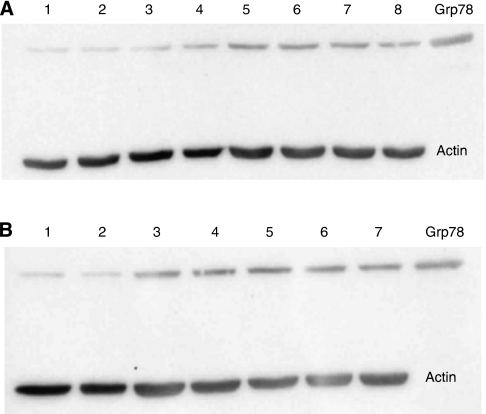
The effect of tamoxifen on T47D cells cultured in oestrogen-supplemented media. Western blots of cancer cell line (T47D) lysates after culture under different conditions. (**A**) Lane plan: 1: standard culture conditions; 2: 0.1% DMSO-supplemented media; 3: 1 nM oestrogen-supplemented media; 4: 1 nM oestrogen plus 1 *μ*M tamoxifen-supplemented media; 5: 1 nM oestrogen plus 5 *μ*M tamoxifen-supplemented media; 6: 1 nM oestrogen plus 10 *μ*M tamoxifen-supplemented media; 7: 1 nM oestrogen plus 15 *μ*M tamoxifen-supplemented media; 8: 1 nM oestrogen plus 20 *μ*M tamoxifen-supplemented media; (**B**) lane plan: 1: standard culture conditions; 2: standard conditions + 0.1% DMSO; 3: 1 *μ*M tamoxifen-supplemented media; 4: 5 *μ*M tamoxifen-supplemented media; 5: 10 *μ*M tamoxifen-supplemented media; 6: 15 *μ*M tamoxifen-supplemented media; 7: 20 *μ*M tamoxifen-supplemented media.

Exposure to a range of tamoxifen doses (1, 5, 10, 15, 20 *μ*M) did not block the UPR activating effect of oestrogen stimulation (Figure 4A). In fact, tamoxifen exposure alone is sufficient to induce UPR activation (Figure 4B).

### *In vitro* cytotoxicity

**Figure 5:**
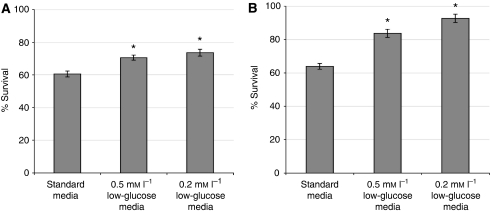
Relative % toxicity for T47D cells under normal and glucose-deprived culture conditions when exposed to previously determined IC_50_ doses of doxorubicin (**A**) and 5-FU (**B**). Relative % survival (mean +/− s.e.m.) for T47D cells under normal and glucose-deprived culture conditions when exposed to 3 *μ*g ml^−1^ doxorubicin (**A**) and 20 *μ*g ml^−1^ 5-FU (**B**). Survival is significantly higher under conditions of low glucose compared with standard culture conditions. Data derived from three triplicate repeats, ^*^P=<0.05.

Under standard culture conditions the IC_50_ (inhibitory concentration 50%) dose of 5-FU was determined to be 20 *μ*g ml^−1^ and for doxorubicin 3 *μ*g ml^−1^ (data not shown). Figure 5 shows that glucose deprivation significantly enhanced resistance to the IC_50_ dose of both 5-FU and doxorubicin when compared with cells cultured under normal glucose conditions (*P*=0.001 for 5-FU and *P*=0.008 for doxorubicin, respectively).

## Discussion

Activation of the UPR has been reported in a wide variety of cancers, and there is extensive evidence for its *in vivo* activation in human tumours, (Fernandez *et al*, 2000; Shuda *et al*, 2003; Uramoto *et al*, 2005; Zhang *et al*, 2006; Scriven *et al*, 2007b; Al-Rawashdeh *et al*, 2008; Scriven *et al*, 2008).

This study is the largest series of breast cancer cases showing UPR activation as shown by upregulation of GRP78 and that a key downstream effector of the UPR, XBP1, is also over-expressed.

The percentage of breast cancers that show UPR induction in our data (78% over-express GRP78) is similar to that shown by other researchers; 67% of breast cancers were found to over-express GRP-78 in a study by Lee *et al* (2006). The present data did not show any association between standard prognostic factors for breast cancer such as grade, nodal status and Nottingham Prognostic Index. This is not surprising given the high percentage of positive expression found and the relative insensitivity of semi-quantitatively scored immunohistochemistry for quantitation of protein levels. More precise study of this association might be provided by mRNA expression measurement using q-PCR.

This study is the first to show an association between both GRP78 and XBP-1 with increasing ER positivity in clinical breast cancer samples. A possible link between oestrogen stimulation and the UPR was first suggested in 1997 using breast cancer cell lines (Kiang *et al*, 1997). Gene expression profiling of *ex-vivo* breast cancer tissue has previously shown an association between ER expression and XBP-1 expression, (Sørlie *et al*, 2001). Fernandez *et al* analysed a small (*n*=25) sample of breast cancers and found over-expression of GRP78 on western blotting in four of six ER-positive tumours and three of three ER-negative tumours. The sample size is too small to confidently assert that there is an association with GRP78 over-expression in ER-negative tumours (Fernandez *et al*, 2000).

There is little current understanding of the link between the two pathways. There is speculation that activation of the UPR may be associated with the development of anti-oestrogen resistance (Fu *et al*, 2007; Gomez *et al*, 2007). UPR induction reduces the occurrence of apoptosis in oestrogen-dependent breast cancer cells treated by oestrogen withdrawal, (which clinically correlates with the use of aromatase inhibitors, Fu *et al*, 2007). Over-expression of XBP-1, another marker of UPR activity, is correlated with resistance to the apoptotic and anti-proliferative effects of anti-oestrogens, (Davies *et al*, 2008). The UPR-activated splice variant of XBP-1, (XBP-1s), confers anti-oestrogen resistance and oestrogen-independent growth in breast cancer cell lines *in vitro* (Gomez *et al*, 2007).

The findings of this study further support a link between the UPR and oestrogenic stimulation. Oestrogenic stimulation in otherwise ideal culture conditions is sufficient to activate the UPR as shown by GRP78 over-expression. These data support the 1997 findings of Kiang and colleagues. The data presented here also extend this by showing for the first time that oestrogenic stimulation is also sufficient to induce downstream effectors of UPR activation such as XBP1. The data shown here are derived from cell culture using phenol red containing RPMI 1640. Phenol red is known to have weak oestrogenic effects. It binds to ERs with an affinity 0.001% of oestradiol and can mask the effects of oestradiol stimulation and the partial agonist effects of some anti-oestrogens such as tamoxifen (Berthois *et al*, 1986). Despite the concerns of phenol red acting as a weak oestrogen the data consistently show that culture under normal control conditions does not result in over-expression of GRP78 or XBP1. Addition of oestradiol to the culture medium at physiological levels shows a consistent over-expression of GRP78 in a dose-dependant manner.

The UPR is known to confer resistance to anti-cancer agents and these data support previous *in vitro* studies, which have shown that topoisomerase II-directed agents such as doxorubicin and etoposide are inhibited by UPR activation (Shen *et al*, 1987; Hughes *et al*, 1989; Chatterjee *et al*, 1995). In clinical studies, a reduced time to recurrence has been linked to over-expression of GRP-78 in women with early breast cancer treated with doxorubicin (Lee *et al*, 2006), so these findings may be of direct clinical relevance.

Our data also show for the first time that UPR induction confers resistance to 5-flurouracil another agent commonly used in the treatment of breast cancer, usually as part of a multi-agent regime.

The UPR is currently a stimulating interest within cancer therapeutics as it is cytoprotective against commonly used anti-cancer agents such as doxorubicin, 5-FU and cisplatin. Agents that target the UPR may be clinically useful in either reducing resistance to the existing anti-cancer drugs or representing a novel therapeutic target in its own right. Further study of the link between the UPR and the development of anti-oestrogen resistance is also needed, to facilitate understanding of why this occurs and provide strategies to prevent it. Again, UPR targeting agents may have a function in this field.
